# The effect of early surgery after hip fracture on 1-year mortality

**DOI:** 10.1186/s12877-015-0140-y

**Published:** 2015-10-28

**Authors:** Paola Colais, Mirko Di Martino, Danilo Fusco, Carlo Alberto Perucci, Marina Davoli

**Affiliations:** Department of Epidemiology, Regional Health Service, Via Santa Costanza 53, 00198 Rome, Italy; Senior Epidemiologist Consultant, Rome, Italy; National Outcome Program, Italian Agency for Health Services, Via Puglie 23, 00187 Rome, Italy

**Keywords:** Hip fracture, Timing surgery, Administrative data, Postoperative complications

## Abstract

**Background:**

Hip fracture injuries are identified as one of the most serious healthcare problems affecting older people. Many studies have explored the associations among patient characteristics, treatment processes, time to surgery and various outcomes in patients hospitalized for hip fracture. The objective of the present study is to evaluate the difference in 1-year mortality after hip fracture between patients undergoing early surgery (within 2 days) and patients undergoing delayed surgery in Italy.

**Methods:**

Observational, retrospective study based on the Hospital Information System (HIS). This cohort study included patients aged 65 years and older who were residing in Italy and were admitted to an acute care hospital for a hip fracture between 1 January 2007 and 31 December 2012. A multivariate Cox regression analysis was used to assess the effect of early surgery on the likelihood of 1-year mortality after hip fracture, adjusting for risk factors that could affect the outcome under study. The absolute number of deaths prevented by exposure to early surgery was calculated.

**Results:**

We studied a total of 405,037 admissions for hip fracture. Patients who underwent surgery within 2 days had lower 1-year mortality compared to those who waited for surgery more than 2 days (Hazard Ratios -HR-: 0.83; 95 % CI: 0.82–0.85). The number of deaths prevented by the exposure to early surgery was 5691.

**Conclusions:**

This study is the first to evaluate the association between time to surgery and 1-year mortality for all Italian elderly patients hospitalized for hip fracture. The study confirmed the previous reports on the association between delayed surgery and increased mortality and complication rates in elderly patients admitted for hip fracture. Our data support the notion that deviating from surgical guidelines in hip fracture is costly, in terms of both human life and excess hospital stay.

**Electronic supplementary material:**

The online version of this article (doi:10.1186/s12877-015-0140-y) contains supplementary material, which is available to authorized users.

## Introduction

Hip fracture injuries are identified as one of the most serious healthcare problems affecting older people. Many studies have explored the associations among patient characteristics, treatment processes, time to surgery and various outcomes in patients hospitalized for hip fracture [1, 2].

Some studies reported that preoperative delay might lead to an increase in mortality and adversely influence other clinical outcomes such as infection and pressure sores [3–6]. Clinical guidelines recommend immediate reparative surgery within 24–48 h of hospital admission [7, 8]. A meta-analysis published in 2010 investigating the effect of surgical delay on mortality at various follow-up times found significantly higher all-cause mortality in patients treated surgically more than 24, 48 and 72 h from admission [9]. A recent meta-analysis published in 2012 found that patients who underwent early surgery had significantly lower odds of death and pressure sores than those whose surgery was delayed (OR 0.74; 95 % CI 0.67 to 0.81; OR 0.48, 95 % CI, 0.38–0.60, respectively) [10].

Despite remarkable benefits of early surgery after hip fracture on outcomes and clinical guideline recommendations, optimal care is not always made available to all eligible patients.

Therefore, the proportion of surgery within 48 h after hip fracture is one of the most frequently used indicators of healthcare quality. Hospitals, and more generally healthcare systems, may be compared on the basis of this indicator, with the implicit assumption that higher proportions reflect more appropriate healthcare practice.

Public reporting of hospital performance has become increasingly common and may influence hospital performance through two related pathways [11, 12]. In the first pathway, patients or general practitioners use performance data to choose better performing providers (which may motivate providers to improve performance). Evidence supporting this pathway is scant [13]. In the second pathway, providers respond to performance data because of professional pride, competitiveness and sensitivity to their reputation among peers and implement internal improvement projects [13, 14].

The proportion of surgery within 2 days after hip fracture is an indicator that measures quality in the orthopaedic specialty and is included in the National Outcome Program, which is currently active in the Italian Health System. This program, introduced in 2010, performs comparative analyses of hospital care, and approximately 130 outcome indicators of inpatient care are evaluated [15]. The results provided by the National Outcome Program are updated every year and are publicly available, including the data analysed in this study [16].

The objective of the present study is to evaluate the difference in 1-year mortality after hip fracture between patients undergoing early surgery (within 2 days) and patients undergoing delayed surgery in Italy.

## Methods

### Data sources

This study is based on information from the Hospital Information System (HIS) [17]. Discharge abstracts for all hospitals are routinely collected by the HIS and contain patient demographic data (gender, age), admission and discharge dates, up to 6 discharge diagnoses (International Classification of Disease, 9^th^ Revision, Clinical Modification [ICD-9-CM]), medical procedures or surgical interventions (up to 6), and status at discharge (alive, dead, transferred to another hospital).

Moreover, the National Tax Registry was used to collect information regarding vital status and out-of-hospital deaths.

### Study population

We conducted a retrospective cohort study of patients aged 65 years and older who were residing in Italy and were admitted to an acute care hospital for a hip fracture (ICD-9-CM diagnosis codes 820.0–820.9 in any position) between 1 January 2007 and 31 December 2012. We excluded admissions of patients:

- hospitalized for hip fracture in the previous 2 years;
- who had multiple significant traumas (DRGs 484–487);
- who had a principal or secondary diagnosis of malignant neoplasm (codes 140.0–208.9) at the index admission (current admission for hip fracture) or at previous hospitalizations during the last 2 years.
- transferred from another acute care hospital;
- died within 2 days of admission.

Patients who died within 2 days of admission were excluded in order to give all subjects the same “probability of exposure” and avoid any kind of time related bias. Additionally, patients with hip fracture who did not undergo surgery were excluded because they had different clinical characteristics and 1-year mortality compared to the surgical patients.

### Exposure

The exposure of interest is the surgery within or after 2 days of hospital arrival (difference between date of surgery and date of admission less than or equal to 2 days). The surgeries were identified by the following ICD-9-CM codes: total or partial hip replacement (codes 81.51, 81.52) and reduction of fracture (codes 79.00, 79.05, 79.10, 79.15, 79.20, 79.25, 79.30, 79.35, 79.40, 79.45, 79.50, 79.55).

### Outcome and follow-up

The outcome under study is 1-year mortality. The follow-up period started two days after admission to give all patients the same opportunity of receiving early surgery and was continued until one year.

### Comorbidities

Risk factors potentially associated with the outcome under study were chosen among the conditions identified in the literature [1, 2, 5]. Comorbidities were identified on the basis of ICD-9-CM codes registered either at the index hospitalization or at previous hospital admissions during the last 2 years [18]. Acute events that occurred during the index admission that could be considered complications of care were not included. Details and ICD-9-CM codes are reported in the Additional file 1.

### Statistical analysis

The proportions of surgery performed within 2 days of hospital arrival were calculated. We stratified the patients into seven age groups: 65–69, 70–74, 75–79, 80–84, 85–89, 90–94 and 95–100 years. The multivariate Cox regression analysis was used to assess the effect of early surgery on the likelihood of 1-year mortality after hip fracture, adjusting for other factors (age, gender and comorbidities) that could affect the outcome under study. In particular, age and gender were considered a priori risk factors and were thus included in the risk-adjustment models. For the co-morbidities, a bootstrap stepwise procedure that assigned an importance rank for the predictors in the logistic regression was implemented to identify the set of conditions that significantly predicted the risk of the outcome and optimized the trade-off between the goodness-of-fit of the final model and parsimony. Using this approach, the logistic regression with all predictors was run 1000 times on random samples drawn with replacement from the original data set. Only the risk factors identified as significant (*p* ≤ 0.05) at least 30 times in at least 30 % of the procedures were included in the predictive model [19]. The adjusted hazard function for both groups of patients (patients with versus patients without early surgery) was calculated. A slight violation of the proportional hazards assumption was detected for the exposure variable (early surgery) using the Schoenfeld Residuals test. We addressed this violation using interactions with time. Therefore, time-dependent effects of early surgery were calculated estimating its effect in the first and in the last six months of follow-up. Time-dependent effects were estimated for the entire population and by age groups.

The absolute number of deaths prevented by exposure to early surgery were calculated. After adjusting the mortality rate among exposed by means of direct standardization, the number of prevented deaths among exposed was calculated as follow: rate among unexposed – adjusted rate among exposed. This absolute effect, referring to one person-year, was subsequently multiplied by the total number of person-years among the exposed.

All analyses were undertaken using SAS Version 9.2 and STATA 12.

The data used for the study are not openly available. The Department of Epidemiology has been authorized by the Ministry of Heath to use the data.

The study was conducted with the permission of the Department of Epidemiology of Lazio Regional Health Service, the regional referral centre for epidemiological research who has full access to anonymized hospitalization data therefore ethics approval was not required.

## Results

**Fig. 1:**
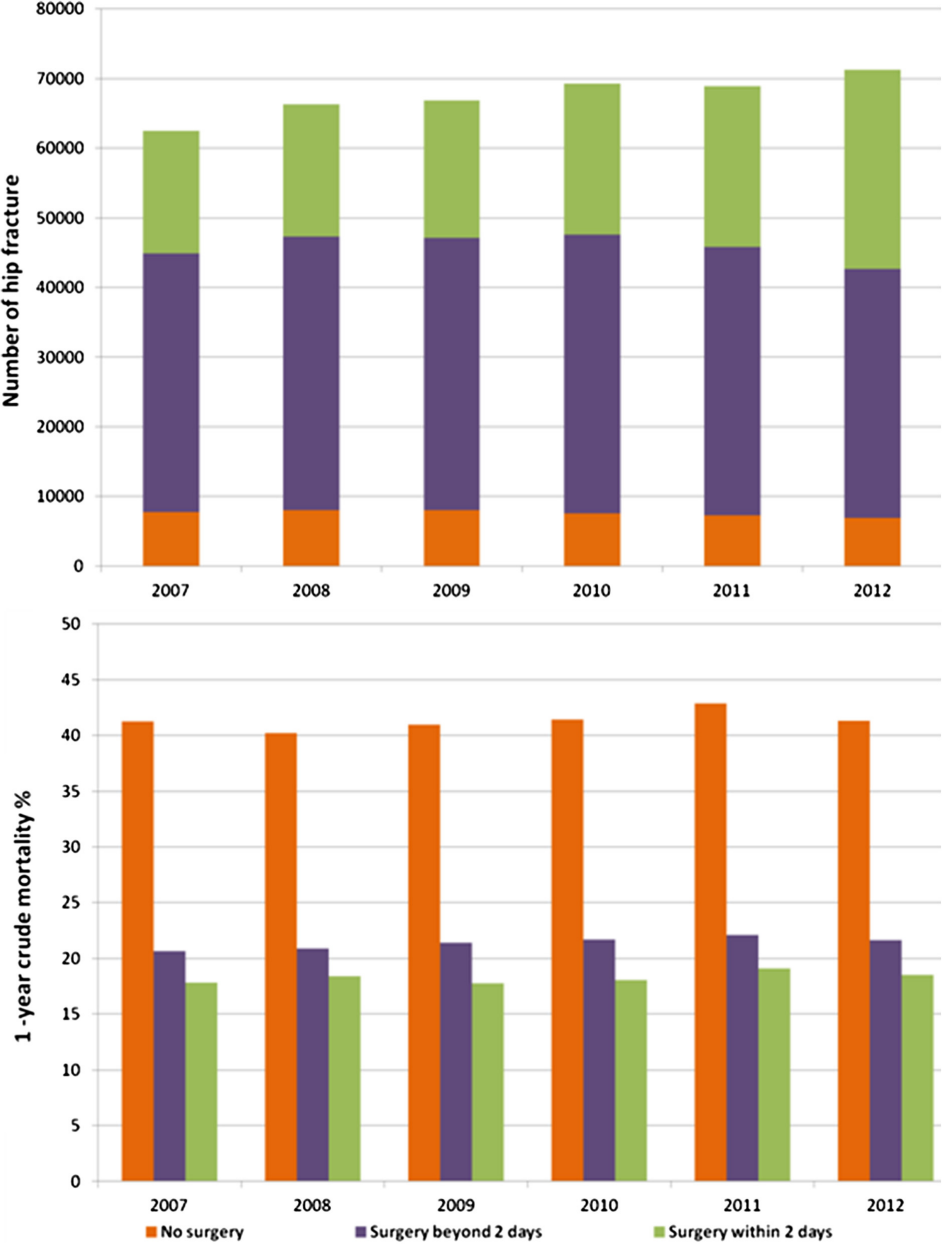
Number of hip fractures and 1-year crude mortality by year and time to surgery

We studied a total number of 405,037 admissions for hip fracture in Italy between 1 January 2007 and 31 December 2012. The number of hip fractures and 1-year crude mortality for time to surgery and year is reported in Fig. 1. Overall, only 11 % had no procedure recorded, the mortality rate of non-operated patients was twice the mortality of the total population, and comorbidities were more frequent in non-operated patients compared with surgical patients. The distribution of risk factors for operated and non-operated patients is reported in Table 1. Therefore, in the further analyses, we only considered 359,529 surgical patients.

**Table 1:** Distribution of risk factors for operated and non-operated patients

Risk factors	Operated patient %	Non-operated patients %
Age groups (years)		
65–69	4.9	4.0
70–74	9.2	8.0
75–79	16.9	15.5
80–84	26.2	24.9
85–89	26.4	26.9
90–94	12.1	14.5
95–100	4.3	6.2
Gender		
Male	21.8	30.2
Female	78.2	69.8
Diabetes	5.9	9.3
Nutritional deficiencies	0.4	1.1
Obesity	0.3	0.4
Blood disorders	4.2	6.9
Dementias including Alzheimer’s disease	8.3	12.5
Parkinson’s disease	2.4	3.0
Hemiplegia and other paralytic syndromes	0.6	0.8
Hypertension	13.0	17.3
Other forms of chronic ischemic heart disease	7.9	13.5
Heart failure	4.9	9.0
Rheumatic heart disease	0.9	1.6
Cardiomyopathy	0.9	2.2
Other heart conditions	0.7	1.0
Cardiac arrhythmias	7.1	10.6
Cerebrovascular disease	10.0	15.7
Vascular disease	2.8	4.8
COPD	4.7	8.4
Chronic renal disease	4.8	9.1
Other chronic disease (liver, pancreas, intestine)	1.9	3.8
Rheumatoid arthritis and other inflammatory polyarthropathies	0.4	0.4

The risk factors included in the predictive model were the following: age, gender, diabetes, nutritional deficiencies, obesity, blood disorders, dementias, Parkinson’s disease, hemiplegia and other paralytic syndromes, hypertension, other forms of chronic ischemic heart disease, heart failure, rheumatic heart disease, cardiomyopathy, other heart conditions, cardiac arrhythmias, cerebrovascular disease, vascular disease, COPD, chronic renal disease, other chronic disease (liver, pancreas, intestine), rheumatoid arthritis and other inflammatory polyarthropathies. All comorbidities included in the predictive model increased the likelihood of 1-year mortality with the exception of hemiplegia and other paralytic syndromes and hypertension. The likelihood of 1-year mortality was reduced in female patients.

**Fig. 2:**
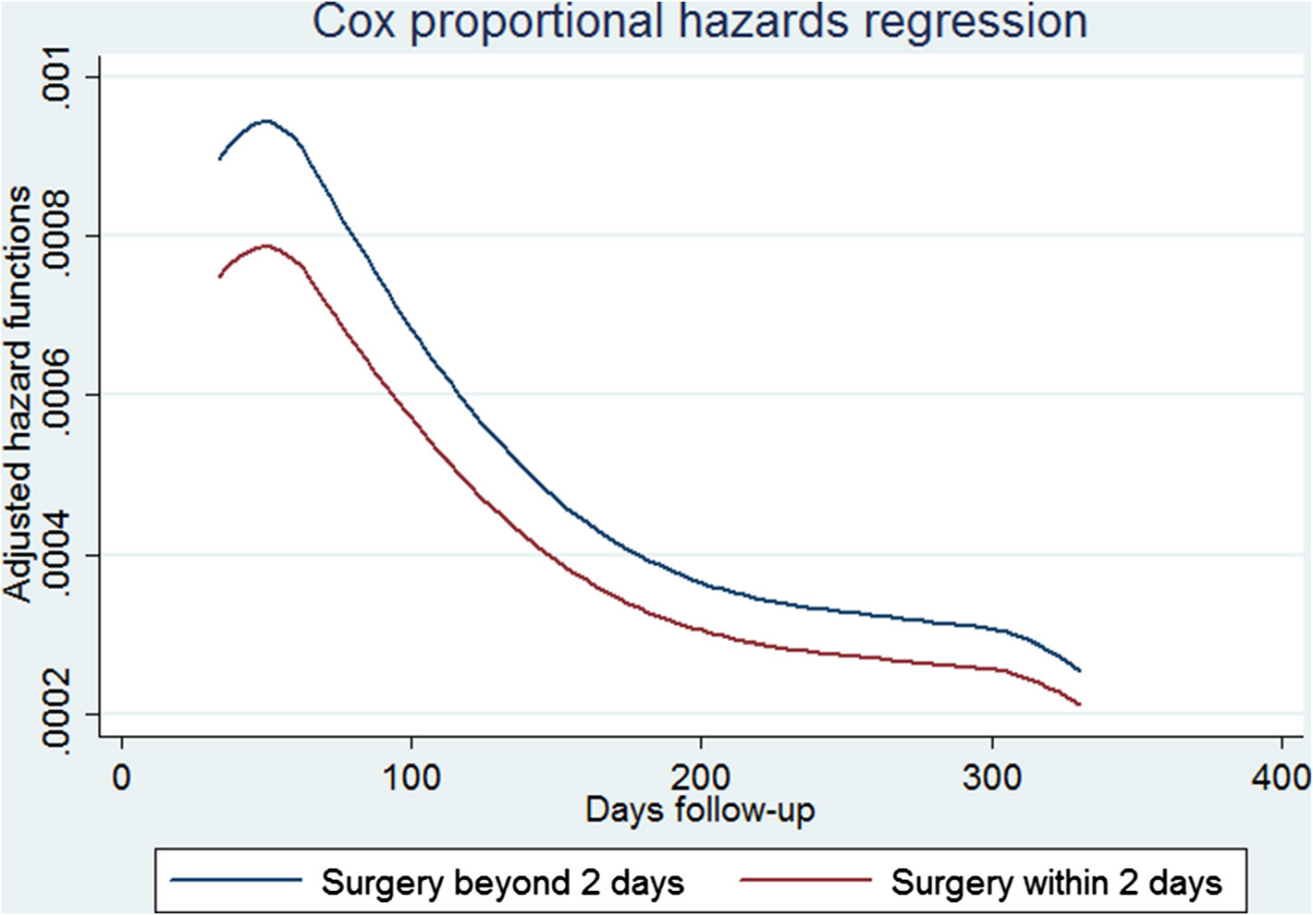
Adjusted hazard functions of 1-year mortality by time to surgery

As shown in Fig. 2, patients who underwent surgery within 2 days had lower 1-year mortality compared to those who waited for surgery more than 2 days (Hazard Ratios (HR): 0.83; 95 % CI: 0.82-0.85). There was a significant interaction between exposure to early surgery and age group (*p* < 0.001); HRs progressively increased with age, ranging from 0.68 (95 % CI: 0.58–0.78) in the age group 65–69 up to 0.88 (95 % CI: 0.83–0.92) in the age group 99–100 (Table 2).

**Table 2:** One-year mortality in patients operated within 2 days versus patients operated beyond 2 days by age group

Age groups (years)	N	H R^a^	95 % C.I.
65–69	17500	0.68	0.58–0.78
70–74	32976	0.72	0.66–0.78
75–79	60697	0.79	0.75–0.84
80–84	94357	0.84	0.81–0.87
85–89	95076	0.84	0.82–0.86
90–94	43468	0.87	0.84–0.90
95–100	15455	0.88	0.83–0.92
Total	359529	0.83	0.82–0.85

^a^Adjusted for age, gender, diabetes, nutritional deficiencies, obesity, blood disorders, dementias, Parkinson’s disease, hemiplegia and other paralytic syndromes, hypertension, other forms of chronic ischemic heart disease, heart failure, rheumatic heart disease, cardiomyopathy, other heart conditions, cardiac arrhythmias, cerebrovascular disease, vascular disease, COPD, chronic renal disease, other chronic disease (liver, pancreas, intestine), rheumatoid arthritis and other inflammatory polyarthropathies

**Fig. 3:**
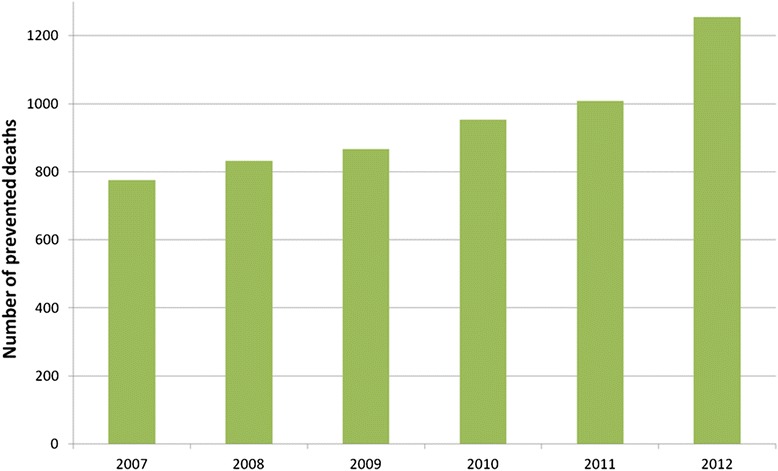
Number of deaths prevented by exposure to early intervention by year

By each age group, the number of deaths observed in the entire cohort of surgical patients, the number of deaths among the exposed (early surgery) and the number of deaths prevented by the exposure among the exposed patients are reported in Table 3. In regard to the population of patients who underwent surgery, the number of deaths prevented by exposure to early intervention was 5691 in the entire follow-up period and increased over time rising from 776 in 2007 to 1255 in 2012 (Fig. 3).

**Table 3:** Number of deaths in the entire population, among the exposed and number of prevented deaths by exposure among the exposed per year by age group

Age groups (years)	N	N of observed deaths in the population	N of observed deaths among the exposed	N of prevented deaths by exposure among the exposed
65–69	17500	967	251	165
70–74	32976	2737	715	387
75–79	60697	7292	2021	696
80–84	94357	17005	5120	1275
85–89	95076	23715	7657	1808
90–94	43468	14759	5340	1017
95–100	15455	6478	2608	460
Total	359529	72953	23712	5691

**Fig. 4:**
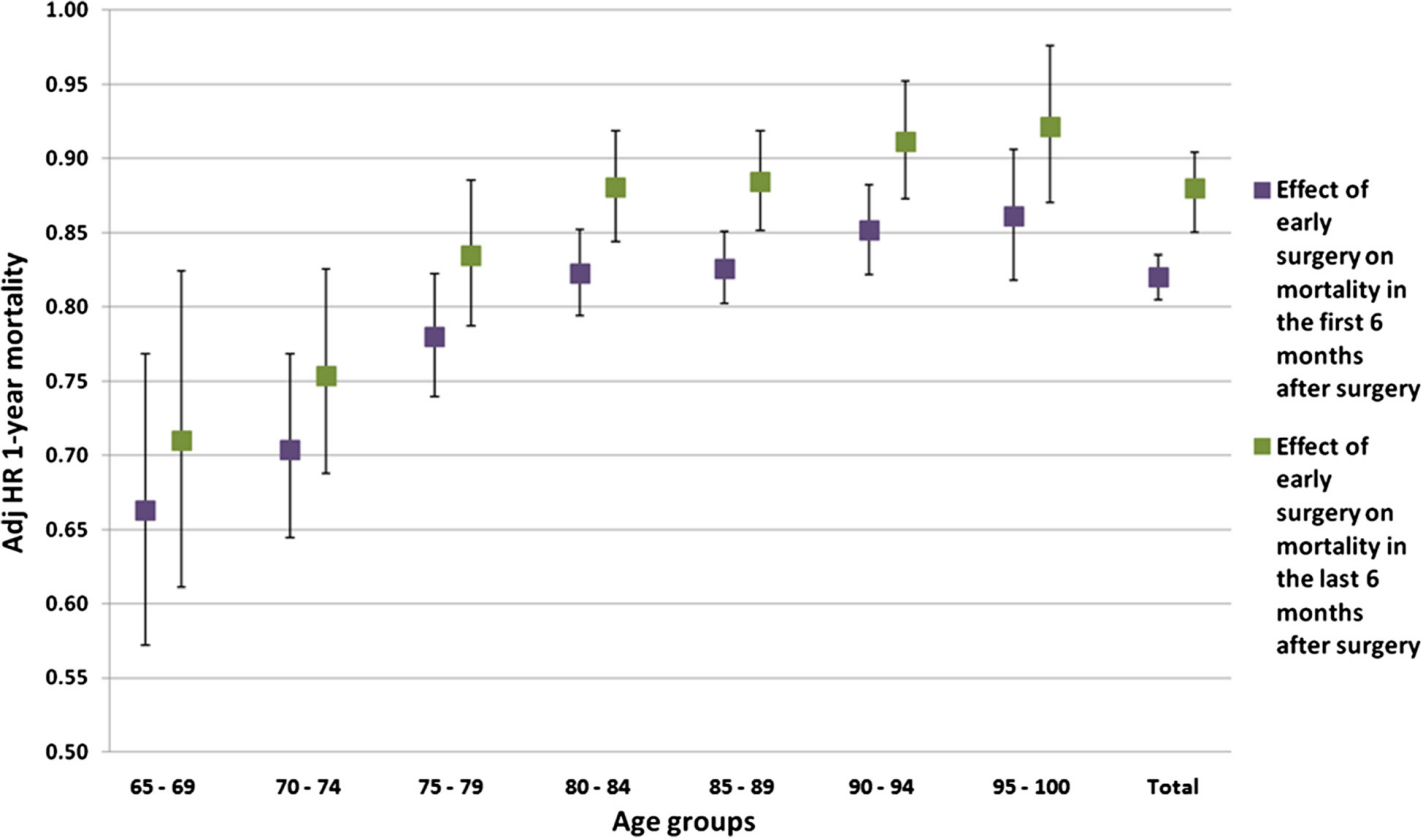
The time-dependent effects of early surgery on mortality by age group

The time-dependent effects (the first 6 months versus the last 6 months of follow-up) of early surgery on 1-year mortality for the entire population of surgical patients and by age groups are shown in Fig. 4. The beneficial effect of early surgery decreased from the first six-month follow-up period to the second six-month period.

## Discussion

The study evaluated the difference in 1-year mortality after hip fracture between patients undergoing early surgery (within 2 days) and patients undergoing delayed surgery in Italy, with the implicit assumption that higher proportions reflect more appropriate healthcare practice.

This study is the first to evaluate the association between time to surgery and 1-year mortality for all Italian elderly patients hospitalized for hip fracture in the recent past, using a large national dataset and routinely collected data on hospital admissions.

We found that patients who underwent surgery within 2 days had lower 1-year mortality compared to those who waited for surgery more than 2 days (HR: 0.83; 95 % CI: 0.82-0.85), conditional on survival to two days after admission and excluding those that did not undergo surgery, even after considering potential confounding factors. The number of deaths prevented by exposure to the early intervention was 5691.

Our data confirm the previous reports on the association between delayed surgery and increased mortality in elderly patients admitted for hip fracture repair. In fact, Simunovic et al. reported a relative risk equal to 0.81 (CI 95 %: 0.68–0.96) whereas Moja et al. an odds ratio equal to 0.74 (CI 95 %: 0.67–0.81) [9, 10].

Novack et al. showed that the length of surgery delay had a gradual effect on increasing both short-term and long-term mortality [4]. Similar findings were also reported from Casaletto and Gatt [20], Zuckerman et al. [21], and Elliott et al. [22].

Delay in hip fracture surgery is significantly associated with increased risk of mortality and complication. Therefore, we support the initiatives to reduce waiting times for hip fracture surgery as a measure to improve local healthcare quality of patients with hip fracture. In Italy, the proportions of surgery within 2 days after hip fracture were included in the National Outcome Program to measure quality in the orthopaedic specialty [23]. However, there are many initiatives in other countries. The Scottish Intercollegiate Guidelines Network suggests that medically fit patients should receive surgery as soon as possible within safe operating hours after presenting to hospital [24]. The British Orthopedic Association guidelines also state that surgical fixation should not be delayed for more than 48 h after admission unless there are clearly reversible medical conditions [25]. The Royal College of Physicians recommends that hip fracture operations should be performed within 24 h by senior staff [26]. As a result, some hospitals, governments, and administrators have set this time point as a target, making hip fracture a performance indicator in the quality of healthcare delivery.

The strengths of this study include the large data sample available for analysis, the validated algorithm for cohort selection and variable definitions, and the robust outcome. However, our study has some potential limitations. Time to surgery was computed based on the dates of hospital admission and surgery and was not refined to the actual hour of surgery and to access in the emergency room, which might introduce a bias into the estimation of the operative delay effect. Emergency room data are not yet available to all Italian regions; however, it was possible to verify the bias introduced in 10 regions for which data were available. For these 10 regions, the proportions of surgery within 2 days were calculated using both the date of admission to hospital and the date of admission in emergency room; in 90 % of cases, no differences in operative delay were observed.

In addition to validating the date of surgery as part of the National Outcome Program, we activated a clinical audit to verify the quality of data reported in the hospital discharge records.

This study relies entirely on administrative data, and despite the broad and valued use as a source for healthcare research, hospital discharge data have several limitations that have been recognized repeatedly [18]. Additionally, although several covariates were included in the models to adjust for differences in patient characteristics, unmeasurable or unmeasured covariates that might affect both the likelihood of 1-year mortality and the delay in surgery after hip fracture, such as cognitive impairment and delirium, may not have been considered. We lacked data on socio-economic status and functional status prior to admission, two factors that may have affected patient selection for surgery, time to surgery, and the outcome of these elderly patients.

## Conclusion

In conclusion, we demonstrated that delaying surgery after hip fracture is associated with worse outcomes. Medical organizations should improve performance according to guidelines and use surgery for hip fracture as a quality improvement measure. Our data support the notion that deviating from surgical guidelines in hip fracture is costly, in terms of both human life and excess hospital stay.

## Additional file

Additional file 1:
**List of comorbidities used for risk adjustment.** (DOC 41 kb)

## References

[1] Roche JJW, Wenn RT, Sahota O, Moran CG (2005). Effect of comorbidities and postoperative complications on mortality after hip fracture in elderly people: prospective observational cohort study. BMJ.

[2] Orosz GM, Magaziner J, Hannan EL, Morrison RS, Koval K, Gilbert M (2004). Association of timing of surgery for hip fracture and patient outcomes. JAMA.

[3] Weller I, Wai EK, Jaglal S, Kreder HJ (2005). The effect of hospital type and surgical delay on mortality after surgery for hip fracture. J Bone Joint Surgery British.

[4] Novack V, Jotkowitz A, Etzion O, Porath A (2007). Does delay in surgery after hip fracture lead to worse outcomes? A multicenter survey. Int J Qual Health Care.

[5] Bottle A, Aylin P (2006). Mortality associated with delay in operation after hip fracture: observational study. BMJ.

[6] Gdalevich M, Cohen D, Yosef D, Tauber C (2004). Morbidity and mortality after hip fracture: the impact of operative delay. Archives Orthopaedic Trauma Surgery.

[7] Mak JC, Cameron ID, March LM (2010). Evidence-based guidelines for the management of hip fractures in older persons: an update. Med J Aust.

[8] Scottish Intercollegiate Guideline Network (SIGN) (2009). SIGN 111: Management of hip fracture in older people.

[9] Simunovic N, Devereaux PJ, Sprague S, Guyatt GH, Schemitsch E, Debeer J (2010). Effect of early surgery after hip fracture on mortality and complications: systematic review and meta-analysis. CMAJ.

[10] Moja L, Piatti A, Pecoraro V, Ricci C, Virgili G, Salanti G (2012). Timing matters in hip fracture surgery: patients operated within 48 h have better outcomes. A meta-analysis and meta-regression of over 190,000 patients. PLoS One.

[11] Fung CH, Lim YW, Mattke S, Damberg C, Shekelle PG (2008). Systematic review: the evidence that publishing patient care performance data improves quality of care. Ann Intern Med.

[12] Berwick DM, Nolan TW, Whittington J (2008). The triple aim: care, health and cost. Health Affairs (Millwood).

[13] Marshall MN, Romano PS (2005). Impact of reporting hospital performance. Qual Saf Health Care.

[14] Davies HT (2001). Public release of performance data and quality improvement: internal responses to external data by US health care providers. Qual Health Care.

[15] Agabiti N, Davoli M, Fusco D, Stafoggia M, Perucci CA (2011). Comparative evaluation of health services outcomes. Epidemiol Prev.

[16] The Italian outcome program. website at http://95.110.213.190/PNEed14/.

[17] Iezzoni LI (1997). Assessing quality using administrative data. Ann Intern Med.

[18] Barone AP, Fusco D, Colais P, D'Ovidio M, Belleudi V, Agabiti N (2009). Effects of socioeconomic position on 30-day mortality and wait for surgery after hip fracture. Int J Qual Health Care.

[19] Austin PC, Tu JV (2004). Automated variable selection methods for logistic regression produced unstable models for predicting acute myocardial infarction mortality. J Clin Epidemiol.

[20] Rogers FB, Shackford SR, Keller MS (1995). Early fixation reduces morbidity and mortality in elderly patients with hip fractures from low-impact falls. J Trauma.

[21] Casaletto JA, Gatt R (2004). Post-operative mortality related to waiting time for hip fracture surgery. Injury.

[22] Zuckerman JD, Skovron ML, Koval KJ, Aharonoff G, Frankel VH (1995). Postoperative complications and mortality associated with operative delay in older patients who have a fracture of the hip. J Bone Joint Surg Am.

[23] Al-Ani AN, Samuelsson B, Tidermark J, Norling A, Ekström W, Cederholm T (2008). Early operation on patients with a hip fracture improved the ability to return to independent living. A prospective study of 850 patients. J Bone Joint Surg Am.

[24] Pinnarelli L, Nuti S, Sorge C, Davoli M, D Fusco, N Agabiti (2012). What drives hospital performance? The impact of comparative outcome evaluation of patients admitted for hip fracture in two Italian regions. BMJ Qual Saf.

[25] Scottish Intercollegiate Guidelines Network (SIGN) (2009). Management of hip fracture in older people. A national clinical guideline.

[26] British Orthopaedic Association Standards for Trauma (BOAST) (2007). Hip fracture in the older person.

